# Proteolytic Processing of Angiotensin-I in Human Blood Plasma

**DOI:** 10.1371/journal.pone.0064027

**Published:** 2013-05-28

**Authors:** Diana Hildebrand, Philipp Merkel, Lars Florian Eggers, Hartmut Schlüter

**Affiliations:** University Medical Centre Hamburg-Eppendorf, Institute of Clinical Chemistry, Mass Spectrometric Proteomics, Hamburg, Germany; Max-Delbrück Center for Molecular Medicine (MDC), Germany

## Abstract

In mammalian species, except humans, N-terminal processing of the precursor peptide angiotensin I (ANG-1-10) into ANG-2-10 or ANG-3-10 was reported. Here we hypothesize that aminopeptidase-generated angiotensins bearing the same C-terminus as ANG-1-10 are also present in humans. We demonstrate the time dependent generation of ANG-2-10, ANG-3-10, ANG-4-10, ANG-5-10 and ANG-6-10 from the precursor ANG-1-10 by human plasma proteins. The endogenous presence of ANG-4-10, ANG-5-10 and ANG-6-10 in human plasma was confirmed by an immuno-fluorescence assay. Generation of ANG-2-10, ANG-3-10 and ANG-4-10 from ANG-1-10 by immobilized human plasma proteins was sensitive to the cysteine/serine protease inhibitor antipain. The metal ion chelator EDTA inhibited Ang-6-10-generation. Incubation of the substrates ANG-3-10, ANG-4-10 and ANG-5-10 with recombinant aminopeptidase N (APN) resulted in a successive N-terminal processing, finally releasing ANG-6-10 as a stable end product, demonstrating a high similarity concerning the processing pattern of the angiotensin peptides compared to the angiotensin generating activity in plasma. Recombinant ACE-1 hydrolyzed the peptides ANG-2-10, ANG-3-10, ANG-4-10 and ANG-5-10 into ANG-2-8, ANG-3-8, ANG-4-8 and ANG-5-8. Since ANG-2-10 was processed into ANG-2-8, ANG-4-8 and ANG-5-8 by plasma proteases the angiotensin peptides bearing the same C-terminus as ANG-1-10 likely have a precursor function in human plasma. Our results confirm the hypothesis of aminopeptidase mediated processing of ANG-1-10 in humans. We show the existence of an aminopeptidase mediated pathway in humans that bypasses the known ANG-1-8-carboxypeptidase pathway. This expands the knowledge about the known human renin angiotensin system, showing how efficiently the precursor ANG-1-10 is used by nature.

## Introduction

The inactive prohormone decapeptide angiotensin I (Ang-1-10) is a key member of the renin angiotensin system (RAS), one of the most important blood pressure and homeostasis regulating systems [1]. ANG-1-10 is released from the circulating preprohormone angiotensinogen by the protease renin. Until today many proteases with the ability to process ANG-1-10 further into angiotensin peptides with different or even opposing physiological actions have been identified. Many of them play a crucial role in the regulation of blood pressure and homeostasis, but are also reported to be involved in other physiological processes like inflammation [2], [3] cell proliferation [4] or the regulation of neuronal processes [5], [6].

The peptide hormone angiotensin II (ANG-1-8) acts as a strong vasoconstrictor but also modulates many other physiological functions by binding to the AT1- or AT2-receptor [1]. ANG-1-8 can be generated by carboxyterminal proteolysis catalyzed by angiotensin converting enzyme-1 (ACE-1) or human mast cell chymase [7]. The vasodilator ANG-1-7 is known to antagonize many physiological effects of ANG-1-8 and can be generated from ANG-1-10 directly [8] as well as from ANG-1-8 and ANG-1-9 [9].

All angiotensin peptides mentioned above are the product of C-terminal cleavage of ANG-1-10. In humans, N-terminal processing of angiotensin peptides by aminopeptidases has only been reported for degradation of ANG-1-8 resulting in the angiotensin peptides ANG-2-8 or ANG-3-8 (also known as AIII and AIV) which are released from ANG-1-8 by aminopeptidase A (APA) and aminopeptidase N (APN) respectively [1]. As regulators of blood pressure both of them play a role in the brain and the central nervous system [10], [11]. The generation of these angiotensin peptides requires the initial C-terminal cleavage of ANG-1-10 by carboxypeptidases like ACE-1 or chymase to form ANG-1-8.

In rats and cats angiotensin peptides deriving from exclusive N-terminal proteolytic cleavage of ANG-1-10 by aminopeptidases were already detected. Such angiotensin peptides contain the same C-terminus as ANG-1-10. The nonapeptide ANG-2-10, octapeptide ANG-3-10 and the hexapeptide ANG-4-10 were described to be generated in the rat [12], [13]. The physiological actions of ANG-3-10 were mainly investigated in the cat [14], [15], [16]. Takai *et al.* found that ANG-5-10 was generated by rat tissues but not by human tissues [17].

In humans little is known about the presence of these angiotensin peptides and their formation to the best of our knowledge. Recently Velez *et al.* proposed that ANG-3-10 is generated proteolytically by human podocytes and showed that ANG-2-10 is generated from ANG-1-10 by human glomerular endothelial cells [18]. The authors also postulated that ANG-3-10 was generated by APN from ANG-2-10. However, it has yet not been found if all of these peptides deriving from aminopeptidase activity are generated by human plasma proteases. Hence here we followed the question whether angiotensin peptides that contain the intact C-terminus of ANG-1-10 are generated in human plasma by aminopeptidases and if these peptides are detectable in blood plasma.

## Materials and Methods

### Ethical Statement

For each condition, volunteers were recruited for this study. According to the requirements of our ethics committee of the medical association Hamburg (Ethikkommission der Ärztekammer Hamburg, Germany) the participants provided signed informed consent.

All procedures concerning the experiments with murine plasma were performed in accordance with protocols approved by the Institutional Animal Care and Research Advisory Committee (Syddansk Universitet Odense, Biomedicinsk laboratorium approval number 157).

### Preparation of Blood Plasma

For incubation experiments human venous citrate blood (ratio 1∶9, blood to sodium citrate 3,13% (Eifelfango)) was obtained by catheterization from the cubital vein of a healthy male volunteer (Age: 50 years, blood pressure: normal, <120/80 mmHg).

For the detection of angiotensin peptides in human plasma and the incubation experiments with ANG-1-10 a volume of 50 ml venous citrate blood was drawn by catheterization from the cubital vein of a healthy female volunteer (Age: 27, blood pressure: normal, <120/80 mmHg). From this blood sample an aliquot of 10 ml was saved for the incubation experiments. The rest of the blood sample was immediately mixed with protease inhibitor cocktail (including 2 mM AEBSF, 0.3 µM aprotinin, 130 µM bestatin, 1 mM EDTA, 14 µM E-64 and 1 µM leupeptin (Sigma-Aldrich)) in a ratio of 1∶50. This sample served for the detection of angiotensin peptides by an immuno-fluorescence assay.

Heparinized (10 IU/ml) mouse and rat blood (200 µl) was obtained from the caudal vein. Plasma was isolated from all blood samples by centrifugation (4–16 K, Sigma) 4000×g for 15 min.

### Immobilization of Plasma Proteins

For the immobilization of plasma proteins CNBr-activated Sepharosebeads® 6MB (GE Healthcare) were used. Protein immobilization was perfomed as described in the product information sheet for CNBr-activated Sepharosebeads® (Sigma Aldrich) except for modifications described in the supporting information part “immobilization of plasma proteins” (Methods S1).

### Incubation of Immobilized Plasma Proteins

Incubation of immobilized plasma proteins and the control samples (glycine derivatized Sepharosebeads® without immobilized proteins and heat inactivated immobilized plasma proteins for the incubation with ANG-1-10) was started by addition of the individual angiotensin peptides (final concentration of 10^−5^ M, solved in HPLC-grade water, Lichrosolve, Merck) to the beads. Incubation was carried out at 37°C on a rotating shaker. The final reaction volume of the samples was 30 µl. At defined incubation times aliquots (3 µl) were taken from the reaction mixtures, diluted in a ratio of 1∶10 in 0.2% (v/v) formic acid/HPLC-grade Water and analyzed by LC-ESI-QQQ-MS (6430 Series, Agilent Technologies) or MALDI-MS (Reflex IV, Bruker).

### Incubation of Immobilized Human Plasma Proteins with ANG-1-10 in the Presence of Protease Inhibitors

Immobilized plasma proteins were separately preincubated for 5 min with the following inhibitors: 200 µM AEBSF (Applichem), 50 µM antipain, 150 µM bestatin, 10 µM captopril, 100 µM chymostatin (Sigma-Aldrich), 100 µM EDTA (Bio-Rad). The control was incubated with immobilized plasma proteins in the absence of inhibitors. The incubation with immobilized plasma proteins was carried out as described in “Incubation of immobilized plasma proteins*”.* The incubation was started by addition of ANG-1-10 to a final concentration of 10^−5^ M to the immobilized plasma proteins.

### Incubation of Non-immobilized Plasma Proteins

For incubation of non-immobilized plasma a reaction volume of 200 µl non-immobilized undiluted and diluted plasma (1∶100 in HPLC-grade water) was used per sample. Incubation was started by addition of ANG-1-10 to a final concentration of 10^−5^ M. Incubation was carried out at 37°C on a rotating shaker. As a control 200 µl of a 10^−5^ M ANG-1-10 solution without plasma was incubated under the same conditions. At defined incubation times (0 h, 0.25 h, 0.5 h, 1 h, 2 h, 4 h, 6 h, 8 h, 24 h) aliquots with a volume of 10 µl were taken from the reaction mixtures and diluted in a ratio of 1∶10 in 0.2% (v/v) formic acid/HPLC-grade Water.

### Peptide Desalting by Solid Phase Extraction

The reaction products derived from the incubation of non-immobilized plasma proteins were desalted by solid phase extraction (Hydophilic lipophilic balance (HLB) µElution plate, Waters). The HLB material was equilibrated by three washing steps with 0.2% formic acid/HPLC-grade water by centrifugation of the plate for 1 min with 500×g. Next, the sample was applied to the HLB material and centrifuged for 2 min with 200×g. The flow-through was discarded and unbound molecules were removed by washing the HLB material 3 times with 0.2% formic acid/HPLC-grade water by centrifugation of the plate for 2 min with 500×g. Adsorbed molecules were eluted with 100 µl 60% MeOH followed by centrifugation for with 200×g. Eluates were collected in a 96 well plate and evaporated to complete dryness in a vacuum concentrator (RC 10, Thermo Scientific). Prior to LC/MS analysis samples were dissolved in 0,2% (v/v) formic acid/HPLC-grade Water.

### Incubation of Recombinant Proteases with Angiotensin Peptides

Each angiotensin peptide (ANG-1-10, ANG-1-8, ANG-2-10, ANG-3-10, ANG-4-10 or ANG-5-10) was dissolved in HPLC-grade water to a final concentration of 10^−5^ M. Incubation was started by addition of 0.25 µg recombinant human aminopeptidase N or angiotensin converting enzyme-1 (ACE-1, R&D Systems) to 100 µl of each angiotensin solution. At defined incubation times aliquots (10 µl) were taken from the reaction mixtures. Reaction was stopped by addition of formic acid adjusting a final concentration of 0.2% (v/v) formic acid/HPLC-grade water. Samples were analyzed by MALDI-MS (Reflex IV, Bruker) using DHB as matrix.

### Mass Spectrometric Peptide Identification and Quantification

Angiotensin peptides were identified by LC-ESI-IT-MS/MS (ion-trap, XCT, Agilent Technologies) or MALDI-MS (Reflex IV, Bruker) and quantified by SRM-coupled ESI-QQQ-MS (Triple quadrupole, 6430 Series, Agilent Technologies). ESI-IT-MS and ESI-QQQ-MS was coupled to an HPLC-chip-system (Agilent Technologies).

Details of the HPLC-chip–MS/MS-system used for analysis are described according to Trusch *et al.* [19]. Individual settings of the system are described in the supporting information part “mass spectrometric identification and quantification” (Methods S2) and in Table S1.

### Detection of Angiotensin Peptides in Human Plasma

The immuno-fluorescence assay was performed as described in the instruction manual (Fluorescent EIA Kit, phoenix pharmaceuticals). For this assay calibration curves of the angiotensin peptides ANG1-10, ANG-4-10, ANG-5-10 and ANG-6-10 were measured using following concentrations (pg/ml): 1, 10, 1000, 10000.

Plasma peptide fractions were obtained by plasma protein precipitation of 15 ml plasma by addition of ACN/0.1% TFA in HPLC-grade water (v/v) in a ratio of 1∶2 (plasma volume:ACN/TFA). The supernatant including the plasma peptides was subjected to a two step chromatographic purification. The first step included a purification by a cartridge filled with HLB material (Oasis HLB cartridge 6 g, Waters). All equilibration, washing and elution steps with this cartridge were carried out as already described for the desalting step of peptides by Oasis HLB-µelution plate in section “Peptide desalting by solid phase extraction”. The eluate containing the desalted plasma peptide fraction was collected in a 50 ml tube. This sample was evaporated to complete dryness and redissolved in 500 µl 0.1% TFA (v/v)/HPLC-grade water. Afterwards a reversed phase chromatography (RP18e, 100 mm×4 mm, Chromolith®performance, Merck KGaA) of the plasma fraction was performed with a 1100 capillary pump (Agilent Technologies) working at 500 µl/min. HPLC-grade water with 0.1% TFA (solvent A) was used for sample loading and delivery. Peptides were eluted from the column using a gradient composed of solvent A and solvent B (acetonitrile) consisting of 3–21% solvent B in 5 min, 21–25% in 40 min, and 25–60% in 1 min.

Eluting fractions were collected with an ÄKTA prime fraction collector (GE Healthcare). Plasma fractions were evaporated to complete dryness prior to the immuno-fluorescence assay. Retention times of angiotensin peptides were determined using synthetic angiotensin peptides under the same chromatographic conditions as described above for the reversed phase chromatography. A volume of 500 µl of the synthetic peptides ANG-1-0, ANG-3-10, ANG-4-10, ANG-5-10 and ANG-6-10 in an equimolar concentration of 10^−5^ M dissolved in 0.1% TFA/HPLC-grade water was loaded onto the column. Separation performance was confirmed by MALDI-MS analysis of the eluted fractions.

Relative fluorescence intensity was measured by 3 flashes and 20 µs integration time with a microplate reader (Infinite m200, Tecan Group Ltd.) with an excitation wavelength of 325 nm (bandwidth 9 nm) and an emission wavelength of 420 nm (bandwidth 20 nm) using optimal gain. Data were processed with i-control software (Version 1.5.14.0, Tecan Group Ltd.) and plotted using Graph Pad Prism Software (Version 4.00).

### Statistics

Data contained in figures with error bars are expressed as mean ± SEM. Statistical analysis was performed using Graph Pad Prism (Version 4.00). Statistical significance was assessed using a one sample 2-tailed student’s *t* test. *P* values less than 0.05 were considered significant.

## Results

### Processing of ANG-1-10 by Immobilized Human and Murine Plasma Proteins

**Figure 1:**
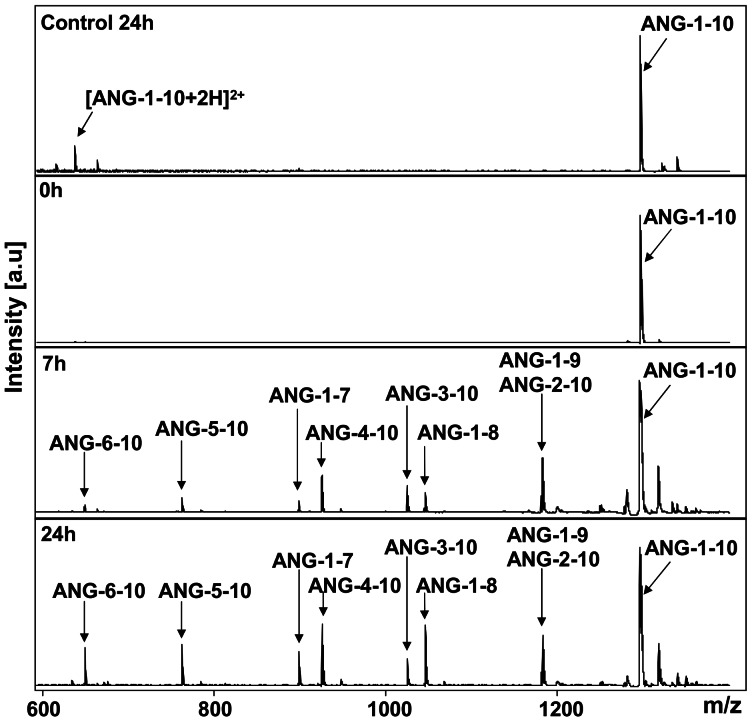
Processing of ANG-1-10 by immobilized human plasma proteins. ANG-1-10 (10^−5^ M) was incubated with immobilized human plasma proteins. Reaction products were detected by MALDI-MS after 0 h, 7 h and 24 h. MALDI-MS signals corresponding to angiotensin peptides are marked by arrows. Control: ANG-1-10 incubated for 24 h with Sepharosebeads® without immobilized proteins.

To investigate the processing of ANG-1-10 by human plasma proteases we used a mass spectrometry based enzyme screening (MES) system [20]. Therefore immobilized murine and human plasma proteins were incubated with ANG-1-10 and the reaction products were analyzed by mass spectrometry. Identification of the reaction products was done by comparison of the m/z values of the peaks in the mass spectra with the theoretical m/z values (Table S2) of possible angiotensin peptide products. ANG-1-10 (m/z 1296.5) was degraded by mouse and rat plasma proteins into the angiotensin peptides ANG-1-8 (m/z 1046.5), ANG-1-7 (m/z 899.5), ANG-2-10 (m/z 1181.7), ANG-3-10 (m/z 1025.6), ANG-4-10 (m/z 926.5), ANG-5-10 (m/z 763.4) (Figure S1+ Figure S2) in a time dependent manner. After 8 h of incubation the only reaction products left were ANG-1-8 after incubation with mouse plasma and ANG-1-7, ANG-1-8 and ANG-5-10 after incubation with rat plasma. All of these peptides were also generated from ANG-1-10 by human plasma proteins, but additionally ANG-6-10 (m/z 650.3) was detected (Figure 1)**.** In addition it was investigated if the processing of ANG-1-10 by immobilized human male plasma proteins, which were used for this experiment, differs from the ANG-1-10-processing by immobilized human female plasma proteins (Figure S3). In comparison to the incubation experiments using male plasma the incubation with immobilized female plasma resulted in the same processing pattern after incubation with ANG-1-10. Neither the control sample containing glycine derivatized Sepharosebeads® without immobilized protein, nor the control sample with immobilized heat inactivated plasma proteins showed significant degradation of ANG-1-10. The identity of the angiotensin peptides generated by human plasma proteins was validated by LC-ESI-IT-MS/MS analysis (Figure S4).

### Chromatographic Purification of Angiotensin Peptides and their Detection in Human Plasma

**Figure 2:**
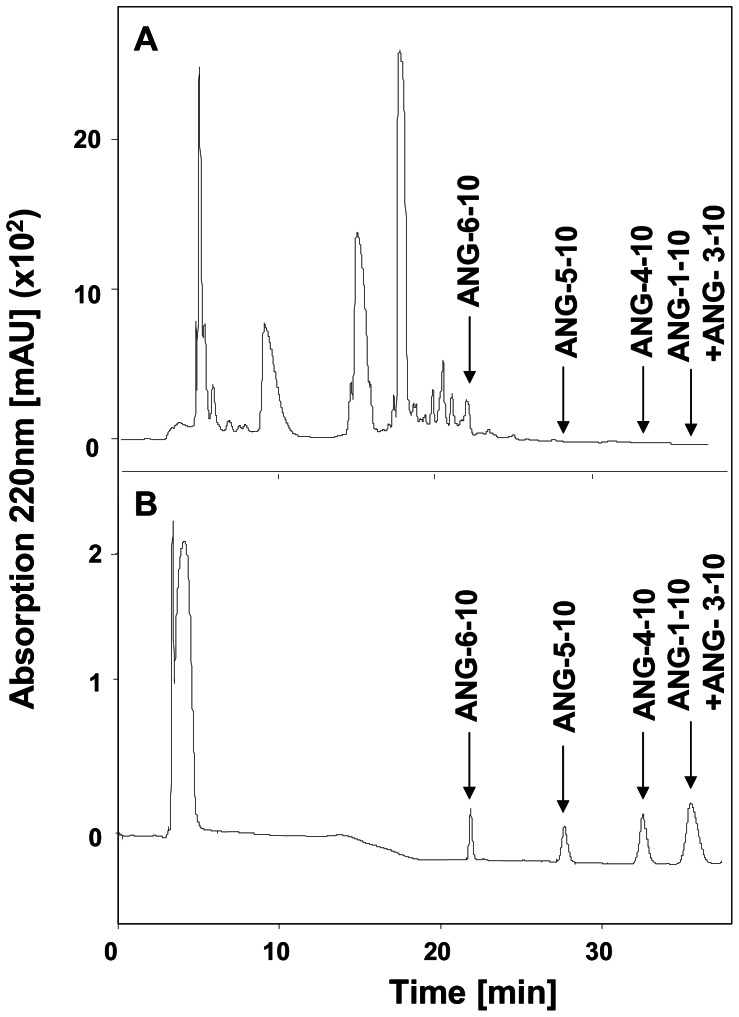
Reversed phase high pressure liquid chromatography (RP-HPLC) of angiotensin peptides. Chromatograms (Absorption at 220 nm plotted against time) after separation of human plasma peptides and synthetic angiotensin peptides by RP-HPLC are shown. A) Chromatogram of the plasma peptide fraction. Retention times of the synthetic angiotensin peptides determined by RP-HPLC of the synthetic angiotensin peptides are indicated by arrows. B) Chromatogram of the synthetic angiotensin peptide mixture including 10^−5^ M ANG-3-10, ANG-4-10, ANG-5-10 and ANG-6-10. Their retention times are indicated by arrows.

To prove whether the peptides bearing the same C-terminus as ANG-1-10 are present endogenously in human plasma we separated the plasma peptides from the plasma proteins by precipitation. Afterwards the plasma peptide fraction was purified by reversed phase high pressure liquid chromatography (RP-HPLC) (Figure 2 A). The separation efficiency and the retention time of the angiotensin peptides ANG-3-10, ANG-4-10, ANG-5-10, ANG-6-10 and ANG-1-10 was determined by RP-HPLC of synthetic angiotensin peptides (Figure 2 B) and subsequent analysis of the derived eluate fractions by MALDI-MS (Data not shown). The angiotensin peptides ANG-4-10, ANG-5-10 and ANG-6-10 were well separated by RP-HPLC and eluted after 32 min, 28 min and 22 min respectively. ANG-1-10 and ANG-3-10 co-eluted after 35 min.

**Figure 3:**
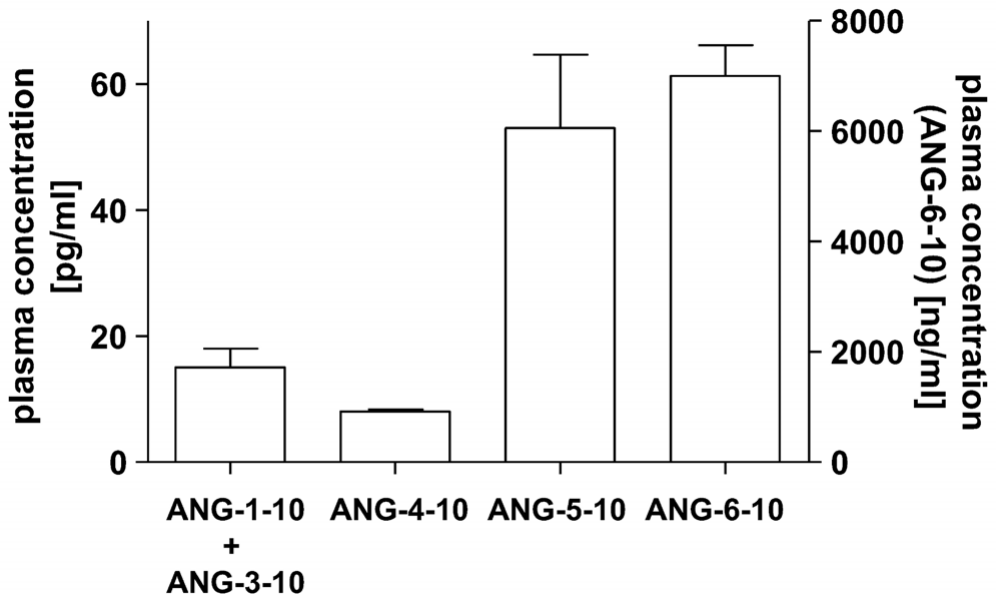
Detection of endogenous angiotensin peptides in human plasma. Concentrations of ANG-1-10+ANG-3-10, ANG-4-10, ANG-5-10 (shown on left y-axis) and ANG-6-10 (shown on right y-axis) were determined by an immuno-fluorescence assay.

The RP-HPLC plasma peptide fractionations corresponding to the retention times of the synthetic angiotensin peptides were analyzed by an immuno-fluorescence assay that was originally manufactured for the quantification of ANG-1-10. The calibration curves of the angiotensin peptides ANG-1-10, ANG-4-10, ANG-5-10 and ANG-6-10 were measured for the quantification of these peptides in human plasma. Using this assay calibration curves were generated for all angiotensin peptides (data not shown) demonstrating the ability of the anti ANG-1-10 antibody (rabbit polyclonal, no cross reactivity with ANG-1-8 or ANG-2-8) to bind angiotensin peptides bearing the same C-terminus. We determined a plasma concentration of about 8 pg/ml for ANG-4-10, 53 pg/ml for ANG-5-10 and 7 ng/ml for ANG-6-10 in the human plasma peptide fractions (Figure 3).

### Processing of ANG-1-10 in Non-immobilized Human Plasma

Plasma protein immobilization is performed by coupling the plasma proteins covalently to CnBR activated Sepharosebeads® via free amino groups of the proteins. Immobilization of proteins stabilizes them because they are covalently fixed and therefore cannot proteolyze other proteins anymore. The hydrophilic environment of the sepharose matrix (agarose) additionally stabilizes proteins since it is comparable with physiological environments. Immobilization usually does not affect the protease activities significantly. In biotechnology immobilization of enzymes is a common approach to stabilize them and to retain their activities and specificities [21].

**Figure 4:**
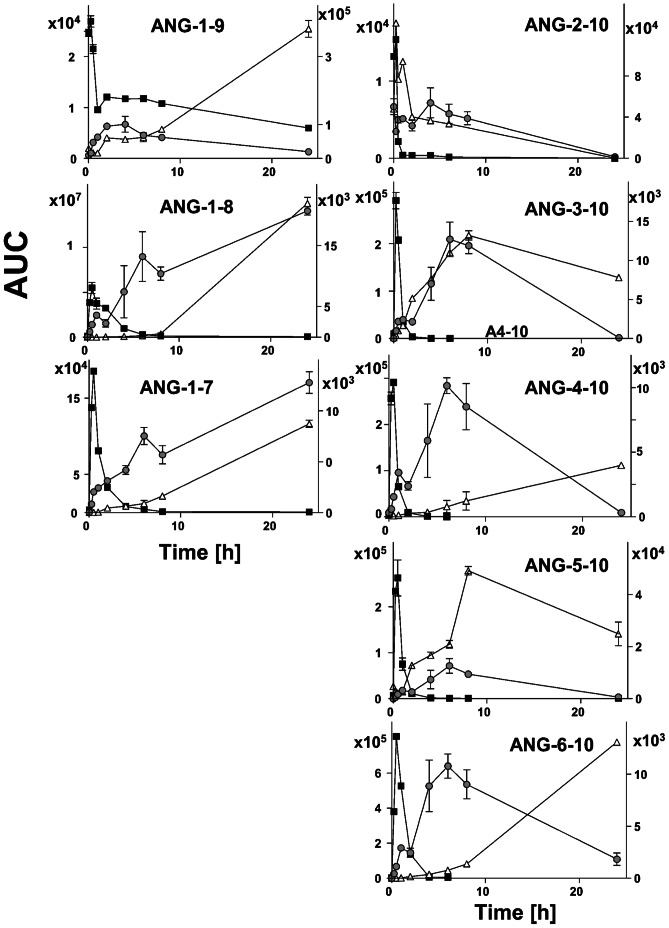
Processing of ANG-1-10 by immobilized, non-immoblized diluted and undiluted human plasma proteins. Immobilized plasma proteins (grey circle), non-immobilized undiluted (closed square) and 1∶100 diluted plasma (open triangle) was incubated with ANG-1-10 (10^−5^ M). Reaction products were analyzed and relatively quantified by SRM-MS. The areas under the curves (AUC) of the reaction products ANG-1-9, ANG-1-8, ANG-1-7, ANG-2-10, ANG-3-10, ANG-4-10 ANG-5-10 and ANG-6-10 are shown. Left y-axis: AUCs of diluted non-immobilized plasma and immobilized plasma (ANG-3-10, ANG-1-9). Right y-axis: AUCs of undiluted non-immobilized plasma and immobilized plasma. Data are shown as mean ± SEM (n = 3, except for ANG-2-10 of the non-immobilized samples with n = 1).

In some cases the *in vitro* generation of angiotensin peptides by immobilized plasma proteins might differ from generation with immobilized plasma proteases due to a change of protease activity caused by removal of protease inhibitors or the dissociation of cofactors through the process of immobilization. To address this question ANG-1-10 was directly incubated with non-immobilized undiluted and diluted human plasma (Figure 4). To compare the angiotensin generating activity of non-immobilized plasma with immobilized plasma, the data from the incubation experiments with ANG-1-10 and immobilized plasma proteins were included in the graphs.

In undiluted plasma the maximum amount of all angiotensin peptides except ANG-1-9 and ANG-2-10 was measured after an incubation time of 0.5 h. The amount of ANG-1-9 and ANG-2-10 showed its maximum after 0 h of incubation followed by a constant decrease, indicating that its generation in undiluted plasma starts within seconds after addition of ANG-1-10 followed by rapid proteolytic degradation. After 0.5 h the amount of the other angiotensin peptides also decreased, indicating their degradation. In diluted plasma samples the amplitude of ANG-2-10 generating activity appeared after 0.25 h. The highest amounts of ANG-3-10 and ANG-5-10 were measured after 8 h, whereas the generation of all other angiotensin peptides ANG-1-9, ANG-1-8, ANG-1-7, ANG-4-10 and ANG-6-10 seemed to be ongoing until the end of the incubation after 24 h. Hence as expected a higher proteolytic ANG-1-10-metabolizing activity was measured in undiluted plasma compared to diluted plasma. The angiotensin generating activity of the immobilized plasma was between the angiotensin generating activity of the non-immobilized undiluted plasma and the 1∶100 diluted plasma. In summary all peptides which have been observed after incubation of ANG-1-10 with immobilized plasma proteins were also generated by incubation with non-immobilized undiluted and diluted plasma in a time dependent manner. It appears that endogenous plasma protease inhibitors do not play a crucial role concerning the proteolytic generation of these angiotensin peptides.

### Incubation of ANG-2-10, ANG-3-10, ANG-4-10 and ANG-5-10 with Immobilized Human Plasma Proteins

**Figure 5:**
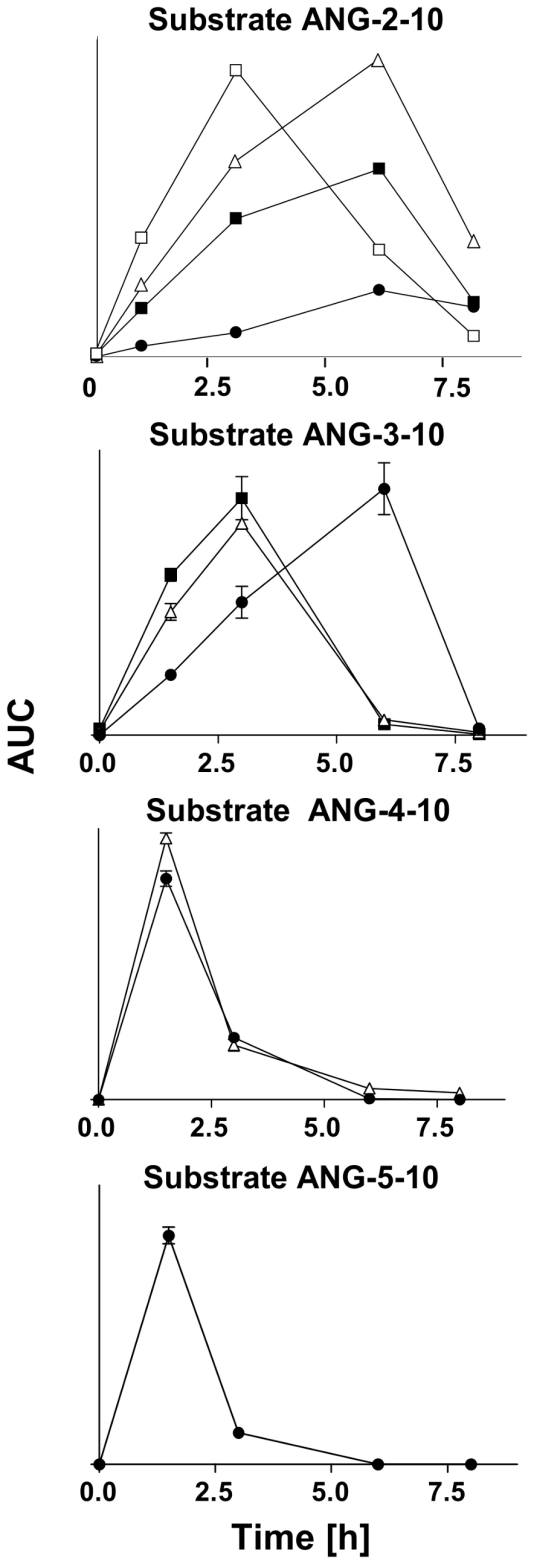
Processing of ANG-2-10, ANG-3-10, ANG-4-10 and ANG-5-10 by immobilized human plasma proteins. Each of these peptides was incubated with immobilized human plasma proteins. Reaction products were analyzed and relatively quantified by SRM-MS. The areas under the curves (AUC) of the generated angiotensin peptides ANG-3-10 (open square), ANG-4-10 (closed square), ANG-5-10 (open triangle), ANG-6-10 (closed circle) are shown (mean ± SEM.). Aliquots were analyzed after 0 h, 2 h, 3 h, 6 h and 8 h (n = 3, except for ANG-2-10 with n = 1).

To investigate if the angiotensin peptides ANG-2-10, ANG-3-10, ANG-4-10, ANG-5-10 and ANG-6-10 are generated successively, each of these angiotensin peptides was incubated with immobilized human plasma proteins. Analysis of the reaction by SRM was performed on a LC-ESI-QQQ-MS system. Exoproteolytic processing of these angiotensin peptides requires the presence of plasma proteases which are able to cognate and cleave the intermediate angiotensin peptides. Incubation of the angiotensin peptide ANG-2-10 with immobilized human plasma proteins lead to the time dependent generation of ANG-3-10, ANG-4-10, ANG-5-10 and ANG-6-10 as was shown by SRM-MS-analysis (Figure 5). The highest amount of ANG-3-10 was generated after 3 h while the maximum amount of ANG-4-10, ANG-5-10 and ANG-6-10 was measured after 6 h. The substrate ANG-3-10 was processed into the peptides ANG-4-10, ANG-5-10 and ANG-6-10 and their maximum amount was generated after an incubation time of 2 h. When ANG-5-10 was incubated in presence of human immobilized plasma proteins time the dependent generation of ANG-6-10 was detected. After an incubation period of 8 h nearly no angiotensin peptides were detected in any of the reaction mixtures. These results demonstrate that, beside ANG-1-10, the intermediate angiotensin peptides ANG-3-10, ANG-4-10, ANG-5-10 serve as substrates for human plasma aminopeptidases.

Analysis of the reaction mixture by MALDI-MS showed, that ANG-2-10 was not only cleaved into the angiotensin peptides ANG-3-10, ANG-4-10, ANG-5-10 and ANG-6-10 but also into the peptides ANG-2-8 (m/z 931.5), ANG-3-8 (m/z 775.4) and ANG-4-8 (m/z 676.3) after 8 h of incubation (Figure S5). The signal for ANG-2-8 had a very low intensity, indicating fast further degradation into ANG-3-8 and ANG-4-8. These peptides were not observed by MALDI-MS after incubation of ANG-3-10 ANG-4-10 or ANG-5-10 (data not shown). This can be seen as an indication that the starting point for the generation of ANG-3-8 and ANG-4-8 likely is ANG-2-8. ANG-2-8 cannot be generated from angiotensin peptides smaller than ANG-2-10 and was also not generated from ANG-1-10 in human plasma. This would also explain why the generation of ANG-2-8, ANG-3-8 and ANG-4-10 was not observed in the reaction mixture after incubation of human plasma with ANG-1-10. Here the generated amount of ANG-2-10 was rapidly converted into the peptides ANG-3-10, ANG-4-10, ANG-5-10 and ANG-6-10 (Figure 1, Figure 4) thus minimizing the availability of ANG-2-10 for its hydrolysis into ANG-2-8. However, ANG-3-10 might also be processed into ANG-3-8 and ANG-4-8 by human plasma proteases. In the same way ANG-4-8 might be generated from ANG-4-10. The generation of these reaction products might be unrecognized due to a fast proteolytic degradation, resulting in low concentrations that might be below the detection limit of our instruments.

### Influence of Protease Inhibitors on the Angiotensin-generating Activity of Immobilized Human Plasma Proteins

Proteases taking part in the aminopeptidase-dependent formation of angiotensin peptides were characterized by incubation of ANG-1-10 with immobilized human plasma proteins in presence of 200 µM AEBSF, 50 µM antipain, 150 µM bestatin, 10 µM captopril, 100 µM chymostatin, 100 µM EDTA and in absence of these inhibitors. Their protease specificity as well as their influences on the angiotensin-generating activity are shown in Table 1. The reaction products were analyzed by SRM-MS after 6 h (Figure S6).

**Table 1:** List of different inhibitors used for characterization of angiotensin-generating proteases.

Inhibitor:	AEBSF	Antipain	Bestatin	Captopril	Chymostatin	EDTA
**Protease specificity:**	Serine	Serine+Cysteine	Amino-peptidases	ACE-1	Serine	Metallo-proteases
**ANG-2-10**	/	−	/	/	/	+
**ANG-3-10**	−	−	/	/	/	+
**ANG-4-10**	/	−	/	/	/	/
**ANG-5-10**	/	/	/	/	/	/
**ANG-6-10**	+	/	+	+	+	−

Influence of inhibitors on generating activity of different angiotensins is indicated by “+” (increase in angiotensin peptide amount) and “–”(decrease in angiotensin peptide amount). Backslash indicates no inhibitory effect.

The generation of ANG-2-10 was inhibited by antipain (−0.12±0.023). The presence of EDTA and bestatin lead to higher ANG-2-10-generating activities. The ANG-3-10-generating activity was inhibited in the presence of AEBSF and antipain. Higher amounts of ANG-3-10 were detected in the presence of bestatin, but a significant raise of ANG-3-10 was only observed in the presence of EDTA (3.4±0.8). The inhibition profile of ANG-4-10 showed many similarities compared to the inhibitor profiles of ANG-2-10 and ANG-3-10 and but a significant inhibition of ANG-4-10-generating activity was only observed in the presence of antipain (-0.21±0.05). The generation of ANG-5-10 was inhibited by AEBSF, antipain and EDTA.

The inhibition profile of the ANG-6-10 generating activity shows clear differences compared to all other angiotensin peptides with the same C-terminus as ANG-1-10. The ANG-6-10-generating activity almost completely disappeared in presence of EDTA (−0.97±0.006) and was slightly inhibited by antipain. Samples including AEBSF (2.80±0.77), bestatin (0.76±0.14), captopril (0.98±0.32) and chymostatin (1.30±0.14) lead to significantly higher amounts of ANG-6-10.

### Aminopeptidase N (APN) as an Angiotensin-generating Protease

APN is a protease that is known to cleave ANG-2-8 between the Arg2-Val3 *in vitro* [22] and *in vivo* [23], [24]. Thus it is a potential protease for the generation of ANG-3-10. To investigate if human plasma APN is able to generate ANG-3-10 and to analyze its substrate and cleavage site specificity recombinant APN (R&D Systems) was incubated with different angiotensin peptides. The reaction products were analyzed by MALDI-MS and the results are summarized in Table 2. After incubation of the APN with ANG-1-10 the reaction product ANG-6-10 was detected after 8 h of incubation. No reaction products were observed after incubation of ANG-1-8 with APN (data now shown).

**Table 2:** Generation of angiotensin peptides from different angiotensin substrates by recombinant aminopeptidase N (APN).

Substrate	ANG-1-10	ANG 1-8	ANG-2-10	ANG-3-10	ANG-4-10	ANG-5-10
Sequence	DRVYIHPFHL	DRVYIHPF	RVYIHPFHL	VYIHPFHL	YIHPFHL	IHPFHL
Cleavage products	HPFHL	none	VYIHPFHL	YIHPFHL	IHPFHL	
			YIHPFHL	HPFHL	HPFHL	HPFHL
			IHPFHL			
			HPFHL			

Sequences of the reaction products which were detected by MALDI-MS are shown.

When ANG-2-10 was used as a substrate for APN the signals of the reaction products ANG-3-10, ANG-4-10 were detected after 1 h of incubation, whereas ANG-5-10 and ANG-6-10 were detected after 4 h and 8 h.

ANG-3-10 was processed into ANG-4-10 and ANG-6-10 by APN within 1 h. The signal for ANG-4-10 was not present in the reaction mixture after longer incubation times. The signal for ANG-6-10 was still present after 24 h, indicating that no further degradation of ANG-6-10 occurred. When ANG-4-10 was incubated with APN, a signal for ANG-5-10 and ANG-6-10 was detected after 1 h of incubation. After an incubation period of 24 h almost no ANG-5-10 was detected, while ANG-6-10 was still present with a high signal to noise ratio.

Incubation of ANG-5-10 lead to the generation of ANG-6-10 whose signal remained stable until 24 h of incubation. No signals of smaller angiotensin reactions products were detected.

### Conversion of Angiotensin Peptides by Recombinant ACE-1

It is known that ACE-1 generates ANG-1-8 by removal of the last two C-terminal amino acids (His9-Leu10) of ANG-1-10. To investigate if ACE-1 can also hydrolyze the angiotensin peptides bearing the same C-terminus as ANG-1-10 we incubated recombinant human ACE-1 with the angiotensin peptides ANG-2-10, ANG-3-10, ANG-4-10, ANG-5-10 and ANG-6-10. The reaction products were analyzed by MALDI-MS (Figure S7). ACE-1 released the two C-terminal aminoacids His9-Leu10 from all angiotensin peptides, except for ANG-6-10. Thus ACE-1 generated the angiotensin peptides ANG-2-8, ANG-3-8, ANG-4-8 and ANG-5-8. This reaction was effectively inhibited by the ACE-1-inhibitor captopril. During incubation of ACE-1 with ANG-6-10 no cleavage product was observed. This is probably due to the small molecular weight (m/z 400.2) of the peptide ANG-6-8, as it consists of 3 amino acids. As a result the signal of ANG-6-8 can be suppressed by interfering signals of DHB-matrix photoproducts which generally occur in a mass range up to 500 Da.

## Discussion

Beside the classical circulating RAS, many other localized RAS as in the brain [25] or the kidney [26] have been discovered. Recently Velez *et al.* investigated the proteolytic processing of ANG-1-10 *in vitro* by human glomerular endothelial cells and podocytes. They showed that ANG-1-10 was proteolytically degraded into ANG-1-8 but was also processed by aminopeptidases. The authors also proposed APA as an ANG-2-10-generating protease and APN as an ANG-3-10-generating protease [18], but still the question remained whether these peptides are also present in human plasma.

Here we show that ANG-1-10 is processed into A-1-9, ANG-1-8, ANG-1-7, ANG-2-10, ANG-3-10, ANG-4-10, ANG-5-10 and ANG-6-10 by immobilized and non-immobilized human plasma proteins (Figure 1, Figure 4, Figure S3 and Figure S4). Concerning the processing pattern of ANG-1-10 no difference could be observed between male (Figure 1) and female plasma (Figure S3). Some of these peptides have already been detected in other species. Velez *et al.* described, that in isolated rat glomeruli ANG-1-10 was mainly converted into ANG-2-10 and ANG-1-7 [27]. Furthermore ANG-2-10 is generated in the hypothalamic extract of rats [28]. ANG-3-10 has been described in the vascular bed of the cat [15], [16], in rat brain and plasma [12] and, as well as ANG-4-10, in rat myocardial tissue [13]. ANG-5-10 has been shown to be generated by rat vascular tissues [17] and in rat peritoneal cell cultures [29]. We did not detect the generation of ANG-6-10 in mouse and rat plasma in contrast to ANG-2-10, ANG-3-10, ANG-4-10 and ANG-5-10 (Figure S1), pointing out that protease activities vary between different species. To the best of our knowledge, no report has described the generation of ANG-6-10 in any species before.

Many proteolytic activities of plasma proteases are strongly regulated by abundant circulating protease inhibitors like alpha-2-macroglobulin, which inhibits a variety of proteases like plasmin, kallikrein and thrombin [30] or C1-inactivator that inhibits proteases of the complement system [31]. In the RAS the ACE-2-activity in plasma was reported to be masked by an endogenous inhibitor that can be chromatographically removed from the protease [32]. With high probability we could exclude that the monitored proteolytic activities are masked by endogenous inhibitors in human plasma which might be removed or dissociated by the process of protein immobilization, since the peptides were also generated in non-immobilized undiluted and diluted human plasma (Figure 4). In comparison to non-immobilized human plasma the only observed effect of plasma protein immobilization was a reduced velocity of the angiotensin generation which was between the angiotensin generating velocity of non-immobilized undiluted and 1∶100 diluted plasma.

We also investigated whether the peptides generated by aminopeptidase activities from ANG-1-10 are present endogenously in human plasma. Indeed, the angiotensin peptides ANG-4-10, ANG-5-10, ANG-6-10 were detected by an immuno-fluorescence assay in human plasma (Figure 3). The generated calibration curves of the angiotensin peptides ANG-1-10, ANG-4-10 and ANG-5-10 indicate cross-reactions of the immuno-fluorescence assay ANG-1-10-antibody with all peptides comprising the same C-terminus as ANG-1-10. Thus we confirm the finding of Velloso *et al.*, who reported about 100% cross reaction of an ANG-1-10-antibody with ANG-3-10 and ANG-4-10 [33]. This finding is crucial concerning the direct measurement of ANG-1-10 in plasma by comparable antibody based assays, as the presence of peptides which derive from N-terminal cleavage of ANG-1-10 might lead to falsified quantification of the examined angiotensin peptide if the plasma peptides are not separated from each other prior to their quantification.

For the same reason we can not exclude that the quantity of ANG-1-10 that was determined by the immuno-fluorescence assay, was not affected by the presence of ANG-3-10 in human plasma, as the synthetic peptides ANG-3-10 and ANG-1-10 coeluted during reversed phase chromatography (Figure 2 A). Thus we prefer to refer to the sum of ANG-1-10 and ANG-3-10 which are present in human plasma with a concentration of 15 pg/ml. Therefore it might be estimated that ANG-3-10 itself is present in plasma in a picomolar concentration range, which is comparable to the finding of Chappell *et al.* who detected ANG-3-10 with a concentration of approximately 30 pg/ml in rat plasma [12]. With 53 pg/ml the ANG-5-10 plasma level we detected is comparable to the plasma levels of ANG-1-8 or ANG-1-7, which are in a range of 20–70 pg/ml [34], [35], [36], [37]. The measured ANG-6-10 concentration of 7 ng/ml is much higher than the levels of ANG-1-8 or ANG-1-7 indicating that ANG-6-10 might be the end product of the catalytic cascade of aminopeptidase activities.

A hint for the successive formation of the angiotensin peptides with the same C-terminus as ANG-1-10 is given by the observation that human plasma aminopeptidase activities are not only able to hydrolyze ANG-1-10 but also ANG-2-10, ANG-3-10, ANG-4-10 and ANG-5-10 resulting in ANG-6-10 as the end product (Figure 5).

Various ANG-1-8-generating proteases like ACE-1, chymase and cathepsin G have been identified until today. In contrast, little is known about ANG-2-10, ANG-3-10, ANG-4-10, ANG-5-10 and ANG-6-10-generating proteases. Here, the proteases taking part in the aminopeptidase-dependent formation of angiotensin peptides were characterized by incubation of ANG-1-10 with plasma in the presence and absence of several protease inhibitors and the reaction products were analyzed by SRM-MS (Table 1, Figure S6).

One question of interest was, if only one protease could be responsible for the generation of all these angiotensin peptides. The similarity between the inhibition profiles of ANG-2-10, ANG-3-10, ANG-4-10 (Table 2, Figure S6) indicates that this might be the case. However, in comparison to the inhibition profiles of these peptides the inhibition profile of ANG-5-10 differed a little, in contrast to the profile of ANG-6-10, which showed remarkable divergence. Thus at least two proteases must be responsible for the generation of these angiotensin peptides. An antipain sensitive protease belonging to the serine or cysteine family must be responsible for the generation of ANG-2-10, ANG-3-10 and ANG-4-10.

APA, a metallopeptidase present in a variety of different tissues and in serum [38], theoretically accounts for the generation of ANG-2-10 as it cleaves ANG-1-8 between the N-terminal amino acids Asp1-Arg2 generating ANG-2-8. But as a metalloprotease EDTA should inhibit APA. Since ANG-2-10 generating activity in human plasma was increased in presence of EDTA APA cannot be responsible for the main part of ANG-2-10 generation. In contrast, EDTA almost completely inhibited the generation of ANG-6-10, whereas ANG-3-10- and ANG-4-10-generating activities increased. This observation clearly shows the existence of an ANG-6-10-generating metalloprotease that differs from the aminopeptidases generating ANG-2-10, ANG-3-10, ANG-4-10. In rat vascular tissue the generation of ANG-5-10 was reported to be completely inhibitable by chymostatin [17]. Formation of the angiotensin peptides ANG-5-10 and ANG-6-10 in human plasma was not inhibited by chymostatin suggesting that the ANG-5-10 generating protease in rat differs from the human one.

ANG-2-8 can be converted into ANG-3-8 by cleavage between the N-terminal amino acids Arg-Val by APN. APN can exist in a membrane bound or soluble state [38], [39] since it was purified from human plasma [40] and urine [41]. Favoloro *et al.* found out that the soluble form of APN owns the predominant functional activity compared to surface associated form [39]. Palmieri *et al.* also showed that APN is able to process ANG-2-10 in cultured porcine aorta endothelium and smooth muscle cells [42]. Taken together APN is a protease that probably also accounts for the generation of ANG-3-10 from ANG-1-10 in plasma.

Incubation of APN with ANG-1-10 resulted in the generation of ANG-6-10 (Table 2). But since long incubation times were necessary to detect its generation ANG-1-10 does not seem to be the preferred angiotensin substrate of APN. ANG-1-8, when used as a substrate itself, was not cleaved by APN (Table 2). This shows that generation of ANG-3-8 requires the initial cleavage of ANG-1-8 into ANG-2-8 by APA.

In contrast, the proteolytic APN-activity on ANG-2-10, ANG-3-10, ANG-4-10, ANG-5-10 and ANG-6-10 was much more distinctive and more effective. Hence we confirm the proposed scheme of Velez *et al.* [18] who suggested the generation of ANG-3-10 from ANG-2-10 by human APN. The same proteolytic pattern could be observed during incubation of ANG-2-10 with human plasma proteins (Figure S5).

Since ANG-6-10 is not further degraded by APN within 24 h it seems to be the stable end product. This might also be an explanation for the high concentration of ANG-6-10 in human plasma that was determined in this work by an immuno-fluorescence assay.

Taken together, APN as a zinc-dependent plasma protease presumably acts as an ANG-6-10-generating protease in human plasma. This would be substantiated by the fact that the ANG-6-10-generating activity was markedly reduced in the presence of EDTA (Table 1, Figure S6). On the other hand the ANG-6-10-generating activity in human plasma was not reduced in the presence of bestatin, a potent APN inhibitor [43], indicating the presence of an additional metalloprotease with ANG-6-10 generating activity.

**Figure 6:**
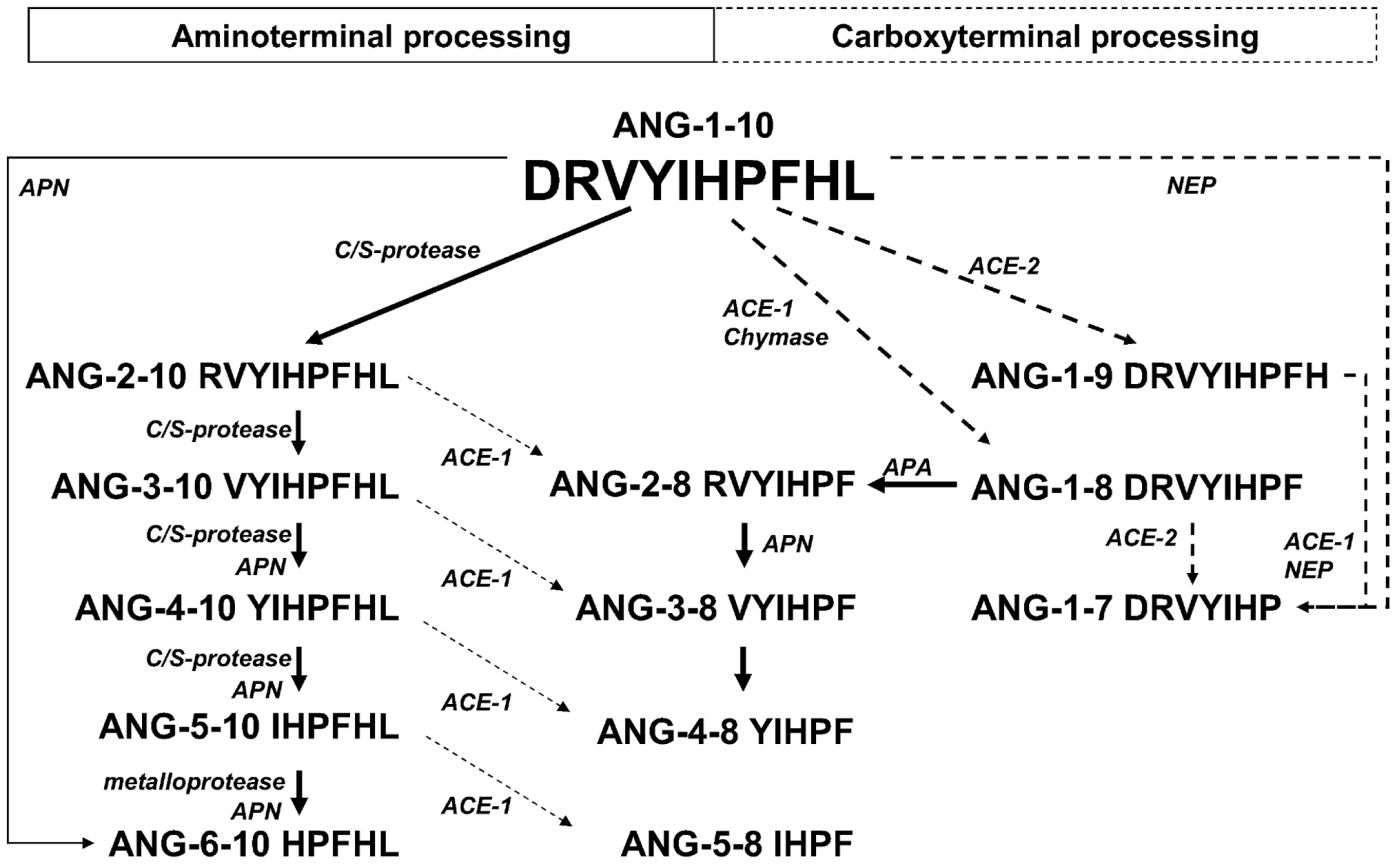
Overview of ANG-1-10 processing by human plasma proteins and by recombinant APN and ACE-1. Arrows with dashed lines indicate hydrolysis from C-terminus. N-terminal-processing is marked by arrows with continuous lines. APN: Aminopeptidase N. C/S-protease: cysteine/serine protease. ACE-1: Angiotensin converting enzyme-1, APA: Aminopeptidase A, NEP: Neprilysin.

The similarities between the generation of angiotensin peptides bearing the same C-terminus as ANG-1-10 in plasma and their generation by APN (Figure 6) also suggest its participation in the generation of ANG-3-10, ANG-4-10 and ANG-5-10 beside an antipain sensitive protease.

The angiotensins bearing the same C-terminus as ANG-1-10 might serve as alternative precursors for the generation of ANG-2-8, ANG-3-8, ANG-4-8, ANG-5-8 and ANG-6-8. Garcia *et al.* proposed that the generation of ANG-2-10 from ANG-1-10 in rat blood depicts a pathway for the proteolytic processing of ANG-1-10 that bypasses the generation of ANG-1-8 [44]. The authors also stated that ACE-1 contributed to the degradation of ANG-2-10 into ANG-2-8 since this conversion was fully inhibited by the ACE-1-inhibitor captopril. It is likely that in humans ACE-1 also catalyzes the removal of the last two C-terminal amino acids His-Leu from ANG-2-10, ANG-3-10, ANG-4-10, ANG-5-10 and ANG-6-10 *in vivo*. This assumption is strongly supported by the results of our incubation experiments were these angiotensins were incubated with recombinant ACE-1 (Figure S7). In this work we show that recombinant human ACE-1 is able to hydrolyze these angiotensin peptides *in vitro.* The incubation of recombinant ACE-1 resulted in the captopril sensitive release of the last two C-terminal amino acids (His-Leu) from ANG-2-10, ANG-3-10, ANG-4-10 and ANG-5-10 finally leading to the generation of the peptides ANG-2-8, ANG-3-8, ANG-4-8 and ANG-5-8.

In Figure 6 an overview is given about the RAS system including the results of this study. Our results demonstrate for the first time the processing of ANG-1-10 by an aminopeptidase-dependent pathway in human plasma which exists in addition to the well known carboxypeptidase pathway. By the aminopeptidase-dependent pathway ANG-2-10, ANG-3-10, ANG-4-10, ANG-5-10 and ANG-6-10 are generated from ANG-1-10. This shows the efficient multi-parallel utilization of the peptide hormone precursor ANG-1-10.

For the generation of the biologically active angiotensin peptides ANG-2-8 and ANG-3-8 the peptides ANG-2-10 and ANG-3-10 can serve as additional substrates, thus bypassing Ang-1-8. Therefore this Ang-1-8 independent pathway can provide both of these physiologically important peptides.

Many physiological effects of ANG-2-8 and ANG-3-8 are similar to the effects of ANG-1-8, including blood pressure regulation by AT1-mediated vasoconstriction [45]. In patients with hypertension the inhibition of ACE-1 by captopril is a common treatment to lower the blood pressure by decreasing the generation of ANG-1-8. Since ANG-2-8 and ANG-3-8 also require the action of ACE-1 captopril is also decreasing the generation of these two vasoconstrictors.

Padia et al. described that ANG-2-8 exerts natriuresis in rats via the AT2-receptor. This effect was not produced by ANG-1-8 [46]. The authors speculated that APN inhibitors might be used to treat diseases characterized by sodium and fluid retention, preventing the degradation of ANG-2-8 to ANG-3-8. This would result in an increase of ANG-2-10, which will not be converted to ANG-3-10 by APN. In addition, this should also increase the generation of ANG-2-8 in the presence of ACE-1. However, according to our results an inhibition of APN will presumably decrease the concentrations of ANG-3-10 and ANG-3-8. As a result side effects may occur, because at least ANG-3-8 [45] is known to exert physiological actions.

Before our study it was generally accepted that the generation of ANG-2-8 and ANG-3-8 goes hand in hand with the hydrolysis of ANG-1-8. Since the regulation of biological processes requires independent control circuits an ANG-1-8-independent pathway for the generation of ANG-2-8 and ANG-3-8 makes sense.

In conclusion our findings demonstrate that the RAS system is equipped with a large number of independently controllable regulator elements which in the future should be investigated in depth regarding the clinical relevance and their impact on drug actions.

## Supporting Information

Figure S1 **Processing of ANG-1-10 by immobilized mouse plasma proteins.** ANG-1-10 (10^−5^ M) was incubated with immobilized mouse plasma proteins. Reaction products were detected by MALDI-MS after 0 h, 3 h, 4 h and 8 h. MALDI-MS signals corresponding to angiotensin peptides are marked by arrows. Control: ANG-1-10 incubated for 24 h with Sepharosebeads® without immobilized proteins.
(TIFF)

Figure S2 **Processing of ANG-1-10 by immobilized rat plasma proteins.** ANG-1-10 (10^−5^ M) was incubated with immobilized rat plasma proteins. Reaction products were detected by MALDI-MS after 0 h, 1 h, 3 h and 8 h. MALDI-MS signals corresponding to angiotensin peptides are marked by arrows. Control: ANG-1-10 incubated for 24 h with Sepharosebeads® without immobilized proteins.
(TIFF)

Figure S3 **Processing of ANG-1-10 by immobilized human female plasma proteins.** ANG-1-10 (10^−5^ M) was incubated with immobilized human female plasma proteins. Reaction products were detected by MALDI-MS after 0 h, 3 h, 4 h and 8 h. MALDI-MS signals corresponding to angiotensin peptides are marked by arrows. Control: ANG-1-10 incubated for 24 h with immobilized heat inactivated plasma proteins.
(TIFF)

Figure S4 **Identification of angiotensin peptides by tandem mass spectrometry.** The reaction products A) ANG-1-9, B) ANG-1-8, C) ANG-1-7, D) ANG-2-10, E) ANG-3-10, F) ANG-4-10, G) ANG-5-10 and H) ANG-6-10 that were generated after incubation of ANG-1-10 with human immobilized plasma proteins were identified by LC/−ESI-IT-MS/MS. Relating MS/MS spectra with assigned b- and y-fragment ions (dashed lines) and the deduced peptide sequence are shown.
(TIFF)

Figure S5 **Processing of ANG-2-10 by immobilized human plasma proteins.** ANG-2-10 (10^−5^ M) was incubated with immobilized human plasma proteins. Reaction products were detected by MALDI-MS after 8 h. MALDI-MS signals corresponding to angiotensin peptides are marked by arrows.
(TIFF)

Figure S6 **Processing of ANG-1-10 by immobilized human plasma proteases in the presence and abscence of protease inhibitors.** ANG-1-10 (10^−5^ M) was incubated with human plasma proteins in presence of 200 µM AEBSF, 50 µM antipain, 150 µM bestatin, 10 µM captopril, 100 µM chymostatin, 100 µM EDTA and absence of any inhibitor. Reaction products were analyzed by SRM-MS after 6 h and the areas under the curves (AUC) of ANG-2-10, ANG-3-10, ANG-4-10, ANG-5-10 and ANG-6-10 from the SRM-MES are shown (mean ± SEM, n = 3). Right y-axis: AUC of ANG-2-10 and ANG-3-10 after incubation in presence of EDTA.
(TIF)

Figure S7 **Processing of angiotensin peptides by recombinant ACE-1.** The angiotensin peptides (10^−5^ M) were incubated with 0.25 µg recombinant human ACE-1 in presence (left panel) and absence of captopril (right panel). ACE-1 was incubated with A) ANG-1-10, B) ANG-2-10, C) ANG-3-10, D) ANG-4-10, E) ANG-5-10. Reaction products were analyzed by MALDI-MS after an incubation time of 8 h. MALDI-MS-Signals which were assigned as angiotensin peptides are marked by arrows.
(TIF)

Table S1 **SRM-transitions and settings for relative quantification of angiotensin peptides by SRM-coupled LC-ESI-QQQ-MS.** Charge states of the precursor ions are denoted in brackets.
(DOC)

Table S2 **Sequences and protonated monoisotopic masses of angiotensin peptides that were generated in human plasma.**
(DOC)

Methods S1 **Immobilization of plasma proteins.**
(DOC)

Methods S2 **Mass spectrometric peptide identification and quantification.**
(DOC)
