# Supplementary figures and images for: Proteolytic Processing of Angiotensin-I in Human Blood Plasma
Source: PLoS One. 2013 May 28;8(5):e64027. doi: 10.1371/journal.pone.0064027 (PMC3665828; doi:10.1371/journal.pone.0064027)

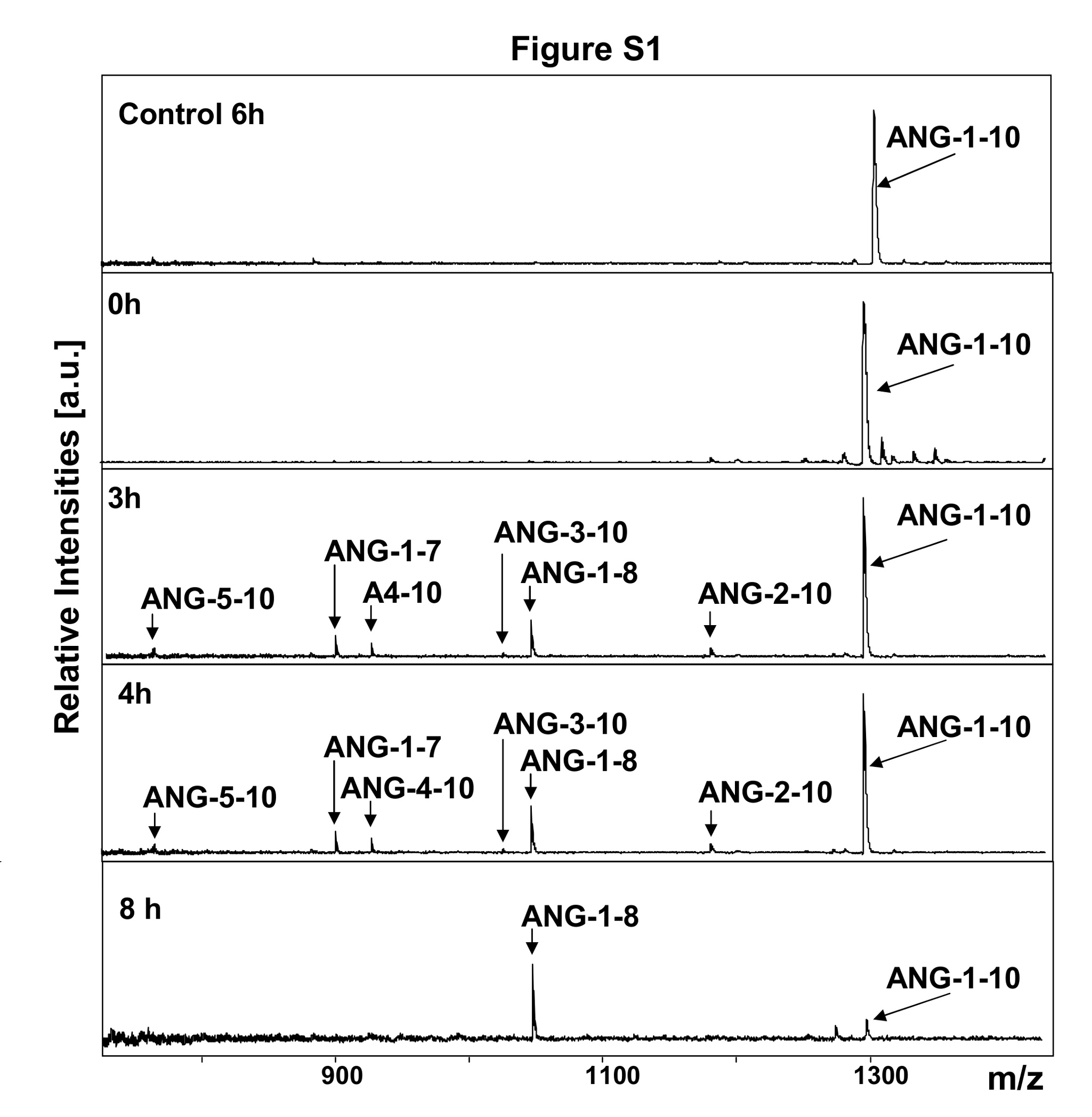

Supplement: Figure S1 — Processing of ANG-1-10 by immobilized mouse plasma proteins. ANG-1-10 (10−5 M) was incubated with immobilized mouse plasma proteins. Reaction products were detected by MALDI-MS after 0 h, 3 h, 4 h and 8 h. MALDI-MS signals corresponding to angiotensin peptides are marked by arrows. Control: ANG-1-10 incubated for 24 h with Sepharosebeads® without immobilized proteins. (TIFF) [file pone.0064027.s001.tiff]
