# Supplementary material for: Proteolytic Processing of Angiotensin-I in Human Blood Plasma
Source: PLoS One. 2013 May 28;8(5):e64027. doi: 10.1371/journal.pone.0064027 (PMC3665828; doi:10.1371/journal.pone.0064027)
Supplement: Table S1 — SRM-transitions and settings for relative quantification of angiotensin peptides by SRM-coupled LC-ESI-QQQ-MS. Charge states of the precursor ions are denoted in brackets. (DOC) [file pone.0064027.s008.doc]

**Supporting Information**

**-Table S1-**

**Table S1. SRM-transitions and settings for relative quantification of angiotensin peptides by SRM-coupled LC-ESI-QQQ-MS.** Charge states of the precursor ions are denoted in brackets.

| **Peptide** | **m/z Precursor ion (charge)** | **m/z**  **Product ion** | **Fragmentor**  **Voltage [V]** | **Collision**  **Energy [V]** |
| --- | --- | --- | --- | --- |
| **A 1-10** | 433 (3+) | 647.0 | 200 | 13 |
| **A 1-10** | 433 (3+) | 269.0 | 170 | 15 |
| **A1-9** | 395.4 (2+) | 619.2 | 135 | 15 |
| **A1-9** | 395.4 (2+) | 156.0 | 135 | 15 |
| **A1-7** | 450.3 (2+) | 647.3 | 135 | 15 |
| **A1-7** | 450.3 (2+) | 619.3 | 135 | 20 |
| **A1-8** | 524 (2+) | 263.2 | 170 | 17 |
| **A1-8** | 392 (3+) | 135.1 | 170 | 15 |
| **A2-10** | 394 (3+) | 532.5 | 135 | 15 |
| **A2-10** | 394 (3+) | 269.3 | 135 | 15 |
| **A3-10** | 342.6 (3+) | 364.0 | 180 | 18 |
| **A3-10** | 342.6 (3+) | 268.0 | 170 | 20 |
| **A4-10** | 309.5 (3+) | 251.0 | 220 | 10 |
| **A4-10** | 309.5 (3+) | 234 | 200 | 12 |
| **A5-10** | 382.2 (2+) | 251.2 | 135 | 10 |
| **A5-10** | 382.2 (2+) | 234.0 | 170 | 13 |
| **A6-10** | 325.6 (2+) | 382.1 | 165 | 7 |
| **A6-10** | 325.6 (2+) | 235.0 | 155 | 8 |
