# Supplementary material for: Proteolytic Processing of Angiotensin-I in Human Blood Plasma
Source: PLoS One. 2013 May 28;8(5):e64027. doi: 10.1371/journal.pone.0064027 (PMC3665828; doi:10.1371/journal.pone.0064027)
Supplement: Table S2 — Sequences and protonated monoisotopic masses of angiotensin peptides that were generated in human plasma. (DOC) [file pone.0064027.s009.doc]

**Supporting Information**

-Table S2-

Table S2. Sequences and protonated monoisotopic masses of angiotensin peptides that were generated in human plasma.

| **Peptide** | **Sequence** | **Monoisotopic peptide mass [M+H]^+^** |
| --- | --- | --- |
| **ANG-1-10** | DRVYIHPFHL | 1296.7 |
| **ANG-2-10** | RVYIHPFHL | 1181.7 |
| **ANG-3-10** | VYIHPFHL | 1025.6 |
| **ANG-4-10** | YIHPFHL | 926.5 |
| **ANG-5-10** | IHPFHL | 763.4 |
| **ANG-6-10** | HPFHL | 650.3 |
| **ANG-1-9** | DRVYIHPFH | 1183.6 |
| **ANG-1-8** | DRVYIHPF | 1046.5 |
| **ANG-1-7** | DRVYIHP | 899.5 |
| **ANG-2-8** | RVYIHPF | 931.5 |
| **ANG-3-8** | VYIHPF | 775.4 |
| **ANG-4-8** | YIHPF | 676.3 |
