# Supplementary material for: Proteolytic Processing of Angiotensin-I in Human Blood Plasma
Source: PLoS One. 2013 May 28;8(5):e64027. doi: 10.1371/journal.pone.0064027 (PMC3665828; doi:10.1371/journal.pone.0064027)
Supplement: Methods S1 — Immobilization of plasma proteins. (DOC) [file pone.0064027.s010.doc]

**Supporting Information**

**Methods S1**

**Immobilization of plasma proteins**

Dry Sepharosebeads® were swollen in 10 ml 0.1 mM hydrochloric acid/ HPLC (high pressure liquid chromatography)-grade water for 30 min on a rotating shaker. Subsequently they were washed repeatedly with HPLC-grade water (Lichrosolve, Merck). Afterwards Sepharosebeads® were equilibrated by multiple washing steps with protein coupling buffer (100 mM NaHCO3, 500 mM NaCl, pH 8,3). For protein immobilization plasma samples with a volume of 40 µl were mixed with coupling buffer in a ratio of 1:6 and mixed with the prepared Sepharosebeads® followed by incubation for 2h on a rotating shaker (room temperature). As a control heat inactivated plasma (boiled for 5 min) was immobilized. A volume of 40 µl swollen Sepharosebeads® was used per sample. After protein immobilization the Sepharosebeads® were washed three times with HPLC-grade water. Free binding sites of the Sepharosebeads® were saturated by incubation with blocking buffer (100 mM NaHCO3,500 mM NaCl, pH 8,3, 1 M Glycine, pH 8,3) for 2h on a rotating shaker (room temperature).

In a addition a sample without immobilized proteins containing only glycine derivatized Sepharosebeads® was prepared as a control for all incubation experiments. Afterwards the blocking buffer was removed by washing repeatedly with HPLC-grade water.
