# Supplementary material for: Proteolytic Processing of Angiotensin-I in Human Blood Plasma
Source: PLoS One. 2013 May 28;8(5):e64027. doi: 10.1371/journal.pone.0064027 (PMC3665828; doi:10.1371/journal.pone.0064027)
Supplement: Methods S2 — Mass spectrometric peptide identification and quantification. (DOC) [file pone.0064027.s011.doc]

**Supporting Information**

**Methods S2**

**Mass spectrometric peptide identification and quantification**

The HPLC-chip contained PLRP-S reversed phase material (300Å, 5 µM). For analysis of angiotensin peptides by LC-ESI-IT-MS 25 µl of the reaction mixture after 24h incubation of immobilized human plasma proteins with ANG-1-10 were injected. Angiotensin peptides were eluted from the RP-sorbent using a linear gradient composed of solvent A (HPLC-grade water/0.2% formic acid) and solvent B (acetonitrile). The gradient consisted of 3–30 % solvent B within 20 min. Nano-electrospray voltage was set at −1850 V. The IT-MS was scanning from 300 to 2000 m/z. Nitrogen at a flow rate of 4 l/min and heated to 325 °C was used as drying gas for spray desolvation. Precursor ion mass spectra were acquired at 0.5 s intervals in positive ion mode, with automated data-dependent MS/MS of the three most intense ions from each precursor MS scan. Doubly charged ions were preferably isolated and fragmented over singly charged ions. The MS/MS experiments were carried out in auto MS/MS mode using a 4 Da window for precursor ion selection. After 3 MS/MS spectra, the precursor ions were actively excluded from fragmentation for at least 1 min. LC-ESI-IT-MS data were analyzed with the software Data Analysis for 6300 Series Ion Trap LC-MS Version 4.0 (Agilent technologies).

The ESI-QQQ-MS which was used for relative quantification was coupled to the same HPLC-chip-System (Agilent Technologies) as described above. A 1200 capillary pump (Agilent Technologies) working at 6 µl/min was used to pump a mixture of solvent A and solvent B, in a ratio of 98:2 for sample delivery (injection volume 6 µl) on the chip enrichment column. A 1200 nano-LC pump (Agilent Technologies) with a flow rate of 400 nl/min was employed for the gradient separation. A linear gradient with 3–30 % of the solvent B over a 11 min was applied. Nanoelectrospray voltage was set at −1750 V. Nitrogen at a flow rate of 3.5 l/min and heated to 350°C was used as drying gas for spray desolvation. Multiple reaction monitoring (MRM)-scan type with the highest resolution mode (Unit) for isolation and fragmentation of the angiotensin precursor ions was used. Precursor ions were fragmented by collision induced dissociation. At least two transitions per angiotensin peptide were scanned and their mass spectra were acquired at 10 ms intervals in positive ion mode. Transitions and optimal settings of fragmentation voltages and collision energies are displayed in Table S1. MRM-Data were analyzed by Mass-Hunter Workstation analysis Version B.03.01 (Agilent Technologies).

For MALDI-MS analysis the matrix 2,5-dihydroxybenzoic acid (DHB, Sigma) solved in 1:1 parts ACN/ 0.2% (v/v) trifluoric acid (TFA) in HPLC-grade water at a final concentration of 30 mg/ml was used. MALDI-MS data were acquired in positive ion reflector mode and analyzed by FlexAnalysis software (Bruker, Version 2.4).
